# Optimized DNA electroporation for primary human T cell engineering

**DOI:** 10.1186/s12896-018-0419-0

**Published:** 2018-01-30

**Authors:** Zhang Zhang, Shunfang Qiu, Xiaopeng Zhang, Wei Chen

**Affiliations:** 1 Laboratory of Vaccine and Antibody Engineering, Beijing Institute of Biotechnology, No. 20, Dongdajie street, Fengtai District, Beijing, 100071 China; 2 Institute of Health Sciences, Anhui University, No. 111, Jiulong Road, Hefei, 230601 China

**Keywords:** Electroporation, T cell engineering, Chimeric antigen receptor modified T cells

## Abstract

**Background:**

Effective gene-delivery systems for primary human T cell engineering are useful tools for both basic research and clinical immunotherapy applications. Pseudovirus-based systems and electro-transfection are the most popular strategies for genetic material transduction. Compared with viral-particle-mediated approaches, electro-transfection is theoretically safer, because it does not promote transgene integration into the host genome. Additionally, the simplicity and speed of the procedure increases the attractiveness of electroporation. Here, we developed and optimized an electro-transfection method for the production of engineered chimeric antigen receptor (CAR)-T cells.

**Results:**

Stimulation of T cells had the greatest effect on their transfection, with stimulation of cells for up to 3 days substantially improving transfection efficiency. Additionally, the strength of the external electric field, input cell number, and the initial amount of DNA significantly affected transfection performance. The voltage applied during electroporation affected plasmid permeation and was negatively correlated with the number of viable cells after electroporation. Moreover, higher plasmid concentration increased the proportion of positively transfected cells, but decreased cell viability, and for single-activated cells, higher cell density enhanced their viability. We evaluated the effects of two clinically relevant factors, serum supplementation in the culture medium and cryopreservation immediately after the isolation of peripheral blood lymphocytes. Our findings showed that our protocol performed well using xeno-free cultured, fresh T cells, with application resulting in a lower but acceptable transfection efficiency of cells cultured with fetal bovine serum or thawed cells. Furthermore, we described an optimized procedure to generate CAR-T cells within 6 days and that exhibited cytotoxicity toward targeted cells.

**Conclusions:**

Our investigation of DNA electro-transfection for the use in human primary T cell engineering established and validated an optimized method for the construction of functional CAR-T cells.

**Electronic supplementary material:**

The online version of this article (10.1186/s12896-018-0419-0) contains supplementary material, which is available to authorized users.

## Background

T cell-based immunotherapy is a promising treatment for cancer, human immunodeficiency virus infection/acquired immunodeficiency syndrome, and other diseases. Current immunotherapies, such as the generation of chimeric antigen receptor (CAR)-T cells or gene editing using clustered regularly interspaced short palindromic repeat (CRISPR) technology, often require the genetic manipulation of T cells [1–3]. A crucial but challenging step involves the introduction of nucleic acids. Conventional delivery techniques include pseudo gamma-retroviral/lentiviral systems and electro-transfection, however, safety, efficiency, and convenience remain important issues in the engineering of human primary T lymphocytes [2, 4–7].

Pseudo gamma-retroviral and lentiviral systems have been widely applied for gene transfer into T lymphocytes and result in an average of 30% to 80% transduction efficiency [8–12]. These viral vectors can integrate into the host genome and lead to permanent transgene expression, with integration-related safety concerns largely resolved during long-term follow-up clinical studies [1, 4]. However, the preparation and transduction of high-quality pseudoviral particles are labor-intensive and time-consuming processes, with large technical requirements. During this procedure, the viral vector is transfected into a package cell line along with two or more package plasmids to generate the pseudoviral particles, which are subsequently purified, concentrated, and titrated before their introduction into T cells. T cells should be activated before viral transduction, and longer culture periods are required for the integration of exogenous genes into the genome and subsequent gene expression [13–16]. This entire process requires from ~ 3 weeks to 1 month.

Electroporation is a faster, theoretically safer, and more economical method relative to viral-particle-based delivery and has been established for T cells [7, 17]. Moreover, the development of other genomic integration techniques, such as the Sleeping Beauty system and CRISPR, has broadened the applications of electroporation [5, 18]; however, systematic studies of electroporation for human primary T lymphocyte engineering have not been performed. In this study, we explored the factors affecting electro-transfection outcomes and developed an optimized DNA electroporation strategy appropriate for T cell engineering and for application in CAR-T cell immunotherapy.

## Methods

### Primary cells and cell lines

Peripheral blood lymphocytes (PBLs) were obtained from two healthy donors, and lymphocytes were harvested via density gradient centrifugation using Ficoll Paque plus (GE Healthcare, Little Chalfont, UK) within 4 h following blood collection. Isolated PBLs were cultured in OpTmizer CTS T-Cell Expansion SFM, 100 IU/mL recombinant human interleukin (IL)-2 (Gibco, Carlsbad, CA, USA), penicillin (100 U/mL) and streptomycin (100 μg/mL), 1% GlutaMAX Supplement, and 5% CTS Immune Cell SR (serum replacement; Gibco) or 10% fetal bovine serum (FBS). PBLs were cryopreserved in Synth-a-Freeze CTS defined protein-free cryopreservation medium (Cell Therapy Systems; Gibco).

CD19-expressing K562 cells (Genechem, Shanghai, China) were used as artificial antigen-presenting cells for the assessment of CAR-T cytotoxicity. Wild type K562 cells (ATCC CCL-243; ATCC, Manassas, VA, USA) was used as non-target cells and cultured in RPMI 1640 medium (Gibco) supplemented with 10% FBS, penicillin (100 U/mL), and streptomycin (100 μg/mL).

### Plasmids and cloning

The pmaxGFP plasmid (Lonza, Cologne, Germany) encoding green fluorescent protein (GFP) was used as an indicator for the assessment of electro-transfection efficiency. The pcDNA3.1(+):19BBz plasmid was constructed by inserting the CAR open reading frame into the multiple cloning site of pcDNA3.1(+). The generated plasmid encoded a second-generation αCD19 CAR containing an FMC63-derived scFv domain and 4-1BB co-stimulation domain. All plasmids were confirmed by sequencing.

### T cell activation and expansion

Fresh or thawed PBLs were cultured at 37 °C in a humidified 5% CO_2_ incubator for 24 h before activation. Cells were suspended at a concentration of 5 × 10^7^ cells/mL and incubated at 20 °C for 30 min with Dynabeads CD3/CD28 CTS (Life Technologies, Grand Island, NY, USA) at a bead-to-cell ratio of 1:3. Cell culture concentration ranged from 5 × 10^5^ to 2 × 10^6^ cells/mL. T cells were re-stimulated at day 11 after isolation, according to a protocol similar to that of the first stimulation, but using a bead-to-cell ratio of 1:1.

### Electroporation of primary T cells

Electroporation was performed using the Celetrix electroporation system (Celetrix, Manassas, VA, USA), according to the following protocol. Cells were counted and suspended with plasmids in the electroporation buffer at cell numbers and plasmid concentrations described in Results section. The resulting mixture containing cells and plasmids was transferred into the pre-cooled Celetrix electroporation cuvette, which was immediately subjected to electroporation. The mixture was then gently transferred to pre-warmed medium and cultured at 37 °C in a humidified atmosphere with 5% CO_2_. T cell cultures containing Dynabeads were removed before cell counting and suspension using magnets (DynaMag portfolio; Life Technologies). We used 20-μL cuvettes to optimize the protocol, resulting in 2 × 10^6^ cells per electroporation corresponding to 1 × 10^8^ cells/mL. Each electroporation experiment was performed in technological triplicate.

### Cell viability assay

Cell viability was analyzed using a Cellometer Auto2000 (Nexcelom Biosciences, Lawrence, MA, USA), an instrument based on acridine orange (AO) and propidium iodide (PI) staining. Uniform cell suspensions (10 μL) were mixed with equal volumes of AO/PI dye (Nexcelom) and subsequently added to the counting board (Nexcelom). Cell viability was calculated using the following formula: viability = the number of live cells after transfection/number of live cells before electroporation. Live cell numbers and diameters were measured by the instrument simultaneously.

### Flow cytometry analysis

Flow cytometry analysis was performed using Guava easyCyte (Merck KGaA, Darmstadt, Germany). Prior to analysis, cells were washed and suspended in phosphate-buffered saline (PBS), containing 1% FBS, and cell concentrations were adjusted to ~ 5 × 10^5^ cells/mL. Data were analyzed using GuavaSoft 3.1 and FlowJoX (TreeStar, Ashland, OR, USA).

For T cells phenotype assay, allophycocyanin (APC)-conjugated anti-human CD3 antibody, PerCP/Cy5.5-conjugated anti-human CD4 antibody, phycoerythrin (PE)-conjugated anti-human CD8 antibody and Alexa Fluor® 488-conjugated anti-human CD62L (Biolegend, San Diego, CA, USA) were used to detect T cells differentiation. APC-conjugated anti-human CD3 antibody and PE-conjugated anti-human CD69 antibody (Biolegend) were used to detect T cells activation. For CAR expression detection, biotinylated-protein L and APC-conjugated streptavidin were used.

### In vitro cytotoxicity assay

A modified version of a flow cytometric cytotoxicity assay was used. Before performing the assay, IL-2 was removed from cell cultures by centrifugation and washing. Target cells were washed and re-suspended in PBS at a concentration of 1 × 10^6^ cells/mL. Fluorescent dye, carboxyfluorescein diacetate succinimidyl ester (CFSE; Invitrogen, Carsbad, CA, USA) was added to the resulting cell suspension at a concentration of 100 nM, followed by incubation at 37 °C for 10 min. The staining reaction was terminated by adding an equal volume of medium containing 10% FBS, and incubating the samples for 10 min at 25 °C, followed by removal of excess CFSE by centrifugation and washing. Labeled target cells were co-cultured with varying numbers of CAR-T cells at 37 °C. The ratio of effector to target cells ranged from 1:1 to 50:1, and for each of these ratios, three technical replicates were analyzed. After a 4 h incubation, 7-aminoactinomycin D was added to the cell suspension at 2 μg/mL, and the viability of target cells was measured by flow cytometry.

### Cytokine enzyme-linked immunosorbent assay (ELISA)

To measure cytokine production, CAR-T cells were cultured with target cells or non-target cells in duplicate wells of a 96-well round-bottom plate for 20 h. Following incubation, supernatants were collected and centrifuged to remove particulates. IL-2, interferon (IFN)-γ, and tumor necrosis factor (TNF)-α were detected by ELISA kits (Valukine Val110, Val104, and Val105, respectively; R&D Systems, Minneapolis, MN, USA). Results were calculated according to manufacturer instructions.

### Statistical analysis

Electro-transfection efficiency data were analyzed using an unpaired two-tailed *t* test with Welch’s correction using GraphPad Prism7 software (GraphPad Software, Inc., San Diego, CA, USA). Results were considered statistically significant at *P* < 0.05, represented by asterisk in the figures. Each experiment comparing influential factors was analyzed using three electro-transfections. Dynamic changes in mean diameter and proliferation were assessed from data collected from three independent experiments.

## Results

### T cell activation improves electroporation efficiency

**Fig. 1:**
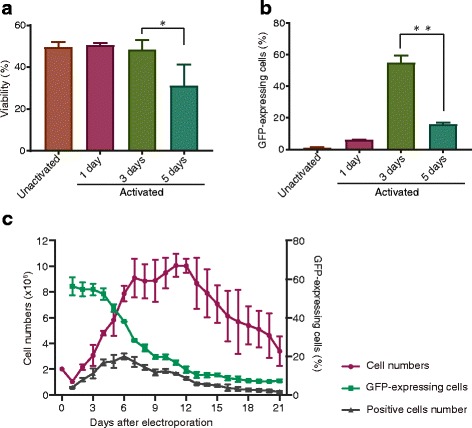
Activation and culturing time affect the efficiency of T cell electroporation. **a**, **b** Cell viability and percentage of positively transfected cells at 24 h after electroporation. **c** Change in the percentage of positively transfected cells (green line) and cell proliferation (red line) after electroporation. Positive cell number (gray line) = percentage of positive cells × viable cell number. Error bars in all figures represent standard deviation

Activation is a necessary step for the expansion of primary T cells in vitro [19]. Therefore, we first examined whether T cell activation affects electroporation efficiency. Freshly isolated lymphocytes were incubated with magnetic beads coated with anti-CD3/CD28 antibodies for stimulation. Unstimulated or stimulated cells (2 × 10^6^) after different incubation times (1, 3, or 5 days) were subjected to electroporation using 1 μg of pmaxGFP plasmids. The following electroporation conditions were used: 500 V, square-wave, 20-ms pulse width, and single pulse. Cell viability and the percentage of GFP-positive cells were monitored using a cell counter and flow cytometry, respectively. Results showed that cell viability in all treatment groups decreased at 24 h after electroporation due to cellular damage from electrical shock (Fig. 1a). Unstimulated cells and cells with shorter activation times (1 and 3 days) showed comparable viabilities. Surprisingly, very low electroporation efficiencies were observed with the unstimulated cells (< 5%; Fig. 1b), but the electroporation efficiency increased along with extended activation time. As shown in Fig. 1b, PBLs stimulated for 3 days showed the highest electroporation efficiency (~ 40% of GFP-expressing cells); however, the transfection efficiency and cell viability of cells subjected to longer activation periods (5 days) were reduced. Cell viability was restored starting from day 2, and cells expanded quickly for ~ 7 days of the incubation (Fig. 1c, red line). GFP expression remained stable for 3 days after electroporation, after which the percentage of positive cells gradually decreased, but remained detectable (6–7%; Fig. 1c, green line).

### Optimization of key parameters for T cell electroporation

**Fig. 2:**
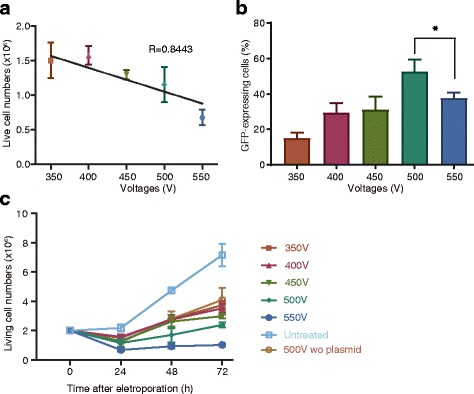
Strength of the externally applied electric field affects primary T cell transfection efficiency, viability, and growth. **a** Changes in viable cell number along with increasing voltage. **b** Transfection efficiency after applying different voltages at 24 h after electroporation. **c** Cell-growth alterations after electroporation

The applied voltage, initial cell number, and plasmid concentration are important factors affecting electro-transfection efficiency. Therefore, we performed a detailed evaluation of their effects on the transfection efficiency of primary T cells. Freshly isolated PBLs (2 × 10^6^) were activated for 3 days and subjected to electroporation using 1 μg of pmaxGFP plasmids under varying voltage conditions (350, 400, 450, 500, and 550 V), and viable cell counts and transfection efficiencies were examined 24 h after electroporation. The results demonstrated that the number of live cells decreased linearly along with increasing voltage in a range of 350 V to 550 V (Fig. 2a). However, the highest transfection efficiency was observed when cells were subjected to electroporation at 500 V (Fig. 2b). Reduced efficiency observed at 550 V might be attributed to cellular damage induced by electric pulses, which was consistent with decreased cell growth at day 3 after electroporation (Fig. 2c).

**Fig. 3:**
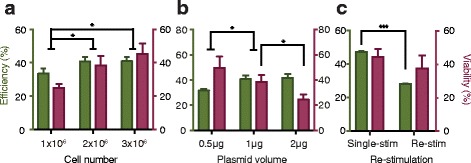
Key factors affecting electro-transfection efficiency. Green bars represent the percentage of positively transfected cells, and purple bars represent cell viability. **a**, **b** Effects of cell number and plasmid input on transfection results. **c** Electro-transfection of re-stimulated T cells. Re-stim, re-stimulated cells; single-stim, single-stimulated cells

Furthermore, we investigated the effects of cell and plasmid input during electroporation. T cells stimulated for 3 days were divided into groups with different cell concentrations (0.5 × 10^8^/mL, 1 × 10^8^/mL, and 1.5 × 10^8^/mL corresponding to 1 × 10^6^, 2 × 10^6^, and 3 × 10^6^ cells per electroporation, respectively) and electroporated using 1 μg of pmaxGFP plasmid. Transfection efficiency and cell viability improved in groups with 2 × 10^6^ and 3 × 10^6^ cells per electroporation as compared with those observed in the 1 × 10^6^ group, whereas no significant differences in cell viability were observed in the 3 × 10^6^ and 2 × 10^6^ groups (Fig. 3a). Activated T cells were then divided into three groups each containing 2 × 10^6^ cells and electroporated with 0.5 μg, 1 μg, or 2 μg plasmids. Flow cytometry results showed that the percentages of GFP-positive cells in the 1-μg and 2-μg groups were higher than that in the 0.5-μg group at 24 h after electroporation. Furthermore, cell viabilities in the 0.5-μg and 1-μg groups were higher than that observed in the 2-μg group (Fig. 3b). These results suggested that a higher plasmid volume during electroporation correlated with an increased percentage of GFP-positive cells, but also with reductions in cell viability.

After initial activation, T cells gradually returned to a near-resting state. Re-stimulation is a useful approach to long-term T cell culture, such as that required during multiple rounds of genome manipulation. Using re-stimulated cells, we determined electro-transfection efficiency when using different DNA and cell input settings at a fixed voltage of 500 V. As shown in Additional file 1: Fig. S1, we found that transfection with 0.5 μg, 1 μg, and 2 μg plasmid resulted in an increased transfection efficiency in the 2 × 10^6^ and 3 × 10^6^ groups, although loss of cell viability along with increased plasmid level was observed in many groups. When DNA input increased to 3 μg, both efficiency and viability decreased severely. Higher cell number might slightly enhance cell viability, as statistically significant differences were only observed in some groups. Generally, electroporation using a higher number of cells yielded more positively transfected cells.

Re-stimulated T cells showed a decreased transfection efficiency relative to single-stimulated T cells at the same experimental settings: 2 × 10^6^ cells, 1 μg plasmid, and 500 V electric field (Fig. 3c). We then determined the mean diameter of T cells, given that electroporation theory indicates that transfection efficiency correlates with cell radius [20]. The radii of live cells increased after activation of T lymphocytes and decreased during the following days of expansion when the cells were returning to the resting state. Unexpectedly, T cell radii increased again after re-stimulation, with similar mean diameters observed in cells stimulated and re-stimulated for 3 days (Additional file 2: Fig. S2).

### Influence of clinical application-associated factors

**Fig. 4:**
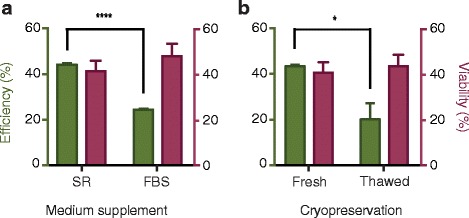
Influence of clinical factors, including serum and cryopreservation, on electroporation efficiency. **a** SR and FBS represent cells cultured with corresponding medium supplement. **b** Fresh and Thaw represent fresh or thawed cells

For the production of clinical-grade T cells, serum used for in vitro T cell culturing is crucial, because it might be contaminated with adventitious pathogens, thereby making it current good manufacturing practice non-compliant [21]. In previous studies, human serum or FBS was commonly used for T cell culturing; however, in the present study, we used xeno-free formatted commercial SR (CTS Immune Cell SR) and examined whether cells cultured with SR or FBS could be transfected using the same electroporation protocol. Freshly isolated PBLs were cultured and activated in media containing 5% SR or 10% FBS for 3 days. The results showed that transfection efficiency in the SR group was considerably higher than that observed in the FBS group, whereas no significant differences in cell viability were observed between these two groups (Fig. 4a).

To the best of our knowledge, there are no previous reports describing the influence of cryopreservation on electroporation. The availability of fresh blood cells is an issue in T cell engineering-related clinical applications; therefore, we tested our electroporation protocol on cryopreserved cells. PBLs were isolated and subsequently cryopreserved at − 80 °C for at least 2 weeks, and cells were thawed and recovered 1 day before activation. Both fresh and cryopreserved cells were activated for 3 days, after which electroporation was performed. We observed that the transduction efficiency of thawed cells was lower, and electroporation did not result in increased damage to the cells (Fig. 4b).

### Rapid generation of CAR-T cells using electroporation

**Fig. 5:**
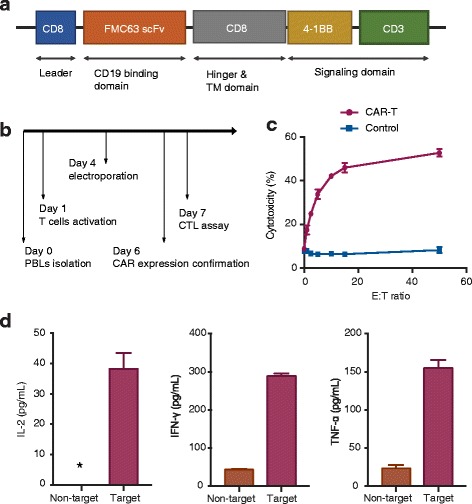
Optimized protocol for the generation of functional CAR-T cells within 1 week. **a** CAR molecular design. Leader, leader peptide; TM, transmembrane domain. **b** CAR-T cell production flowchart. **c** Cytotoxicity of CAR-T cells. E:T ratio represents the ratio of effector and target cells. The red line represents CAR-T cells, and the blue line represents control T cells. **d** Cytokine production of CAR-T cells. Non-target represents the group of wild-type K562 cells and CAR-T cells. Target represents the group of CD19-expressing K562 cells and CAR-T cells. *The IL-2 production of non-target cells groups was too low (< 3.9 pg/mL) to be calculated by a four-parameter logistic standard curve

We then assessed the efficacy of the electroporation method for construction of CAR-T cells. Plasmids encoding the CAR molecule 19BBz targeting human CD19 and containing 4-1BB and CD3ζ stimulatory domains (Fig. 5a) were electroporated into single-activated T cells, and CAR expression was confirmed by protein L staining and flow cytometric analyses at day 6 (Fig. 5b and Additional file 3: Fig. S3a). T lymphocytes expressing 19BBz CAR displayed cytotoxicity toward target cells and produced IL-2, IFN-γ, and TNF-α (Fig. 5c and d).

### T cells represent the predominant cell population at day 2 after electroporation

We used PBLs as the starting material and cultured the cells in OpTmizer CTS T-Cell Expansion SFM, which was specifically developed for the growth and expansion of primary human T lymphocytes. To determine the proportion of T lymphocytes after culturing, we measured the percentage of T cells, revealing increases in this population to 97% on the second day after electroporation (Additional file 3: Fig. S3c).

### The influence of electroporation on T cell differentiation and activation

To investigate the influence of electroporation on T cell differentiation, we detected the proportion of naïve T cells and central-memory T cells, which exhibit high levels of surface CD62L expression [11]. As shown in Additional file 4: Fig. S4, electroporation had little effect on CD4^+^/CD62L^+^ T cells, but reduced the percentage of CD8^+^/CD62L^+^ T cells. Because our electroporation protocol included a stimulation procedure, we also detected dynamic changes in CD69, an indicator of T cell activation [22, 23], with CD69 expression in electroporated T cells lower relative to that in T cells not undergoing electroporation. These results also showed that CD69 expression was significantly elevated after 3 days of stimulation, followed by downregulation on day 4.

## Discussion

In this study, we systematically investigated in vitro human T cell engineering by electroporation, primarily for clinical applications involving immunotherapy. Many commercial electroporation methods have been designed using different mechanisms. Unlike virus-based transduction, various forms of human primary T cell electro-transfection methods exist [7, 18, 24–26]. Therefore, we did not focus on specific parameter settings, but rather investigated common factors that can affect transfection efficiency. We showed that the stimulation of cells is critical to transfection efficiency, and discussed several key parameters and investigated the effects of two clinical factors, SR and cryopreservation, on the outcome of electroporation. Cytotoxicity assays showed that the optimized method developed here was suitable for the rapid production of CAR-T cells. Furthermore, compared with T cells engineered using lentiviral-particle methods, which require ~ 3 weeks, our protocol allowed the generation and analysis of candidate CAR-T cells within 6 to 7 days.

To the best of our knowledge, this is the first primary human T cell transduction study to employ non-viral-based gene-delivery and xeno-free formulation without human- or animal-derived components. The proposed protocol demonstrated the potential for CAR-T cell research and clinical applications. Additionally, we observed that cryopreservation of cells or the culturing of cells with FBS reduced transduction efficiency, but to an acceptable level.

Investigation of the effects of the input cell number and the initial amount of DNA on the transfection results was conducted following determination of the optimal electroporation voltage. In contrast to the parameter settings, which were dependent upon the electric pulse generator, these two factors exerted general and independent effects on transfection. During the electroporation of both initially stimulated and re-stimulated cells, an increase in plasmid input led to higher transfection efficiency and reduced cellular viability. We also found that, generally, higher cell concentration generally yielded more positively transfected cells. Additionally, total cell number and cell concentration were not separately discussed, because the cuvettes and capillaries used in most electroporation instruments have specifically recommended total input volumes pertaining to the mixture of cell and genetic material. Therefore, changes in cell number and concentration were simultaneous.

Following initial stimulation, T cells proliferate for a number of days and can be expanded several hundred-fold by re-stimulation [19, 21]. Our results demonstrated that re-stimulated cells can be efficiently used for electro-transfection. Therefore, when long-term culturing or large cell number is required, T cells can be engineered using re-stimulated cells. Because re-stimulated T cells yield a lower percentage of positively transfected cells using the same experimental settings, further adjustments, such as an increase in cell number and/or plasmid input, are required in order to achieve similar numbers of positive cells and/or efficiency to those obtained using single-activated cells.

During electroporation, the cell radius is negatively correlated with the external electric field required for transfection [20]. The mean diameter of T cells increases following activation, decreases during expansion, and increases again after re-stimulation. Consistent with these findings, we observed a high transfection efficiency in the activated T cells and poor viability of cells stimulated for 5 days; however, different outcomes were observed when using single-stimulated and re-stimulated cells with similar radii. These results suggested that both physical profiles and changes in cell size during stimulation and expansion can partially contribute to transfection efficiency. Previous studies showed the controversial effects of stimulation of human T cells on electro-transfection [7, 17]. Under our experimental conditions, unstimulated T cells were extremely unstable and showed poor viability.

Differentiation profile and activation status have important relationships with T cell capacity and proliferation. For CAR-T therapy, one strategy to enhance their antitumor efficacy involves initiating T cell engineering using defined composition subsets. Early differentiated subsets, such as naïve T cells and central-memory T cells, show improved potency in vivo [11, 27]. Our results showed that electroporation reduced early differentiated subsets in CD8^+^ cells, which might have decreased CAR-T antitumor efficacy in vivo, and lower CD69 expression might be an indicator of poorer proliferation of electro-transfected cells as compared with untreated cells.

CAR-T cells are remarkably efficient according to many clinical and pre-clinical trials investigating hematologic malignancies [1]; however, this therapy induces severe adverse effects due to on-target/off-tumor effects or cytokine-release syndrome, which are difficult to restrain because of constitutive transgenic CAR expression [28, 29]. By contrast, T cells transiently expressing CAR are self-limiting and more governable, which is a particular advantage for application of novel CARs and where clinical safety remains to be thoroughly determined [1, 2, 26]. Moreover, the protocol described in this study can also be used for genome manipulation using CRISPR or transposon systems.

It should be noted that because variations in human primary cells critically influence gene-delivery efficiency, the percentages associated with electro-transfection efficiency using different cells were not constant. A similar issue was previously reported in transductions using viral systems [9]. Nevertheless, the effect of factors investigated in the present study were identical. The transfection efficiency of re-stimulated cells shown in Fig. 3c and those under similar experimental settings in Additional file 1: Fig. S1 were not equal; however, when compared with cells isolated from the same time and donor, single-stimulated T cells still displayed higher transfection efficiency than re-stimulated cells (Fig. 1c), which was a result consistent with that shown in Fig. 3c.

## Conclusion

In this study, we investigated several parameters affecting the electro-transfection efficiency of human primary T cells for immunotherapeutic applications, and an optimized electroporation method for T cell engineering was established. Functional CAR-T cells were successfully produced in 1 week using this protocol. This method can be utilized in combination with other genetic manipulation systems for T cell engineering.

## Additional files

Additional file 1: **Figure S1.** Electroporation of re-stimulated T cells at 500 V. (PDF 804 kb)

Additional file 2: **Figure S2.** Changes in the mean diameter and the growth of T cells after activation. (PDF 736 kb)

Additional file 3: **Figure S3.** Transgene expression and proliferation of CAR-T cells. (PDF 793 kb)

Additional file 4: **Figure S4.** Change of T cell phenotype after electroporation, initial stimulation, and re-stimulation. (PDF 1080 kb)

## Abbreviations

AO: Acridine orange
CAR: Chimeric antigen receptor
CFSE: Carboxyfluorescein diacetate succinimidyl ester
CRISPR: Clustered regularly interspaced short palindromic repeats
ELISA: Enzyme linked immunosorbent assay
FBS: Fetal bovine serum
GFP: Green fluorescent protein
IFN-γ: Interferon-γ
IL-2: Recombinant human interleukin-2
PBLs: Peripheral blood lymphocytes
PBS: Phosphate-buffered saline
PI: Propidium iodide
SR: Serum supplementation
TNF-α: Tumor necrosis factor-α

## References

[1] Fesnak AD, June CH, Levine BL (2016). Engineered T cells: the promise and challenges of cancer immunotherapy. Nat Rev Cancer.

[2] Levine BL (2015). Performance-enhancing drugs: design and production of redirected chimeric antigen receptor (CAR) T cells. Cancer Gene Ther.

[3] June CH, Levine BL (2015). T cell engineering as therapy for cancer and HIV: our synthetic future. Philos Trans R Soc Lond B Biol Sci.

[4] Gill S, June CH (2015). Going viral: chimeric antigen receptor T-cell therapy for hematological malignancies. Immunol Rev.

[5] Ren J, Liu X, Fang C, Jiang S, June CH, Zhao Y (2017). Multiplex genome editing to generate universal CAR T cells resistant to PD1 inhibition. Clin Cancer Res.

[6] Singh H, Figliola MJ, Dawson MJ, Olivares S, Zhang L, Yang G, Maiti S, Manuri P, Senyukov V, Jena B (2013). Manufacture of clinical-grade CD19-specific T cells stably expressing chimeric antigen receptor using Sleeping Beauty system and artificial antigen presenting cells. PLoS One.

[7] Chicaybam L, Sodre AL, Curzio BA, Bonamino MH (2013). An efficient low cost method for gene transfer to T lymphocytes. PLoS One.

[8] Milone MC, Fish JD, Carpenito C, Carroll RG, Binder GK, Teachey D, Samanta M, Lakhal M, Gloss B, Danet-Desnoyers G (2009). Chimeric receptors containing CD137 signal transduction domains mediate enhanced survival of T cells and increased antileukemic efficacy in vivo. Mol Ther.

[9] Kochenderfer JN, Dudley ME, Feldman SA, Wilson WH, Spaner DE, Maric I, Stetler-Stevenson M, Phan GQ, Hughes MS, Sherry RM (2012). B-cell depletion and remissions of malignancy along with cytokine-associated toxicity in a clinical trial of anti-CD19 chimeric-antigen-receptor-transduced T cells. Blood.

[10] Dull T, Zufferey R, Kelly M, Mandel RJ, Nguyen M, Trono D, Naldini L (1998). A third-generation lentivirus vector with a conditional packaging system. J Virol.

[11] Sommermeyer D, Hudecek M, Kosasih PL, Gogishvili T, Maloney DG, Turtle CJ, Riddell SR (2016). Chimeric antigen receptor-modified T cells derived from defined CD8+ and CD4+ subsets confer superior antitumor reactivity in vivo. Leukemia.

[12] Frigault MJ, Lee J, Basil MC, Carpenito C, Motohashi S, Scholler J, Kawalekar OU, Guedan S, McGettigan SE, Posey AD (2015). Identification of chimeric antigen receptors that mediate constitutive or inducible proliferation of T cells. Cancer Immunol Res.

[13] Rosenblum MD, Way SS, Abbas AK (2016). Regulatory T cell memory. Nat Rev Immunol.

[14] Chang JT, Wherry EJ, Goldrath AW (2014). Molecular regulation of effector and memory T cell differentiation. Nat Immunol.

[15] Yang S, Rosenberg SA, Morgan RA (2008). Clinical-scale lentiviral vector transduction of PBL for TCR gene therapy and potential for expression in less-differentiated cells. J Immunother.

[16] Tumeh PC, Koya RC, Chodon T, Graham NA, Graeber TG, Comin-Anduix B, Ribas A (2010). The impact of ex vivo clinical grade activation protocols on human T-cell phenotype and function for the generation of genetically modified cells for adoptive cell transfer therapy. J Immunother.

[17] Van Tendeloo VF, Willems R, Ponsaerts P, Lenjou M, Nijs G, Vanhove M, Muylaert P, Van Cauwelaert P, Van Broeckhoven C, Van Bockstaele DR (2000). High-level transgene expression in primary human T lymphocytes and adult bone marrow CD34+ cells via electroporation-mediated gene delivery. Gene Ther.

[18] Kebriaei P, Singh H, Huls MH, Figliola MJ, Bassett R, Olivares S, Jena B, Dawson MJ, Kumaresan PR, Su S (2016). Phase I trials using Sleeping Beauty to generate CD19-specific CAR T cells. J Clin Invest.

[19] Levine BL, Bernstein WB, Connors M, Craighead N, Lindsten T, Thompson CB, June CH (1997). Effects of CD28 costimulation on long-term proliferation of CD4+ T cells in the absence of exogenous feeder cells. J Immunol.

[20] Gehl J (2003). Electroporation: theory and methods, perspectives for drug delivery, gene therapy and research. Acta Physiol Scand.

[21] Smith C, Okern G, Rehan S, Beagley L, Lee SK, Aarvak T, Schjetne KW, Khanna R (2015). Ex vivo expansion of human T cells for adoptive immunotherapy using the novel Xeno-free CTS Immune Cell Serum Replacement. Clin Transl Immunology.

[22] Yamashita I, Nagata T, Tada T, Nakayama T (1993). CD69 cell surface expression identifies developing thymocytes which audition for T cell antigen receptor-mediated positive selection. Int Immunol.

[23] Swan DJ, Kirby JA, Ali S (2012). Post-transplant immunosuppression: regulation of the efflux of allospecific effector T cells from lymphoid tissues. PLoS One.

[24] Almasbak H, Rian E, Hoel HJ, Pule M, Walchli S, Kvalheim G, Gaudernack G, Rasmussen AM (2011). Transiently redirected T cells for adoptive transfer. Cytotherapy.

[25] Zhao Y, Zheng Z, Cohen CJ, Gattinoni L, Palmer DC, Restifo NP, Rosenberg SA, Morgan RA (2006). High-efficiency transfection of primary human and mouse T lymphocytes using RNA electroporation. Mol Ther.

[26] Zhao Y, Moon E, Carpenito C, Paulos CM, Liu X, Brennan AL, Chew A, Carroll RG, Scholler J, Levine BL (2010). Multiple injections of electroporated autologous T cells expressing a chimeric antigen receptor mediate regression of human disseminated tumor. Cancer Res.

[27] Singh N, Perazzelli J, Grupp SA, Barrett DM. Early memory phenotypes drive T cell proliferation in patients with pediatric malignancies. Sci Transl Med 2016; 8(320):320ra3. 10.1126/scitranslmed.aad5222 26738796

[28] Brentjens R, Yeh R, Bernal Y, Riviere I, Sadelain M (2010). Treatment of chronic lymphocytic leukemia with genetically targeted autologous T cells: case report of an unforeseen adverse event in a phase I clinical trial. Mol Ther.

[29] Morgan RA, Yang JC, Kitano M, Dudley ME, Laurencot CM, Rosenberg SA (2010). Case report of a serious adverse event following the administration of T cells transduced with a chimeric antigen receptor recognizing ERBB2. Mol Ther.

