# Supplementary material for: Optimized DNA electroporation for primary human T cell engineering
Source: BMC Biotechnol. 2018 Jan 30;18:4. doi: 10.1186/s12896-018-0419-0 (PMC5789706; doi:10.1186/s12896-018-0419-0)
Supplement: Supplementary file 1 — Figure S1. Electroporation of re-stimulated T cells at 500 V. (PDF 804 kb) [file 12896_2018_419_MOESM1_ESM.pdf]

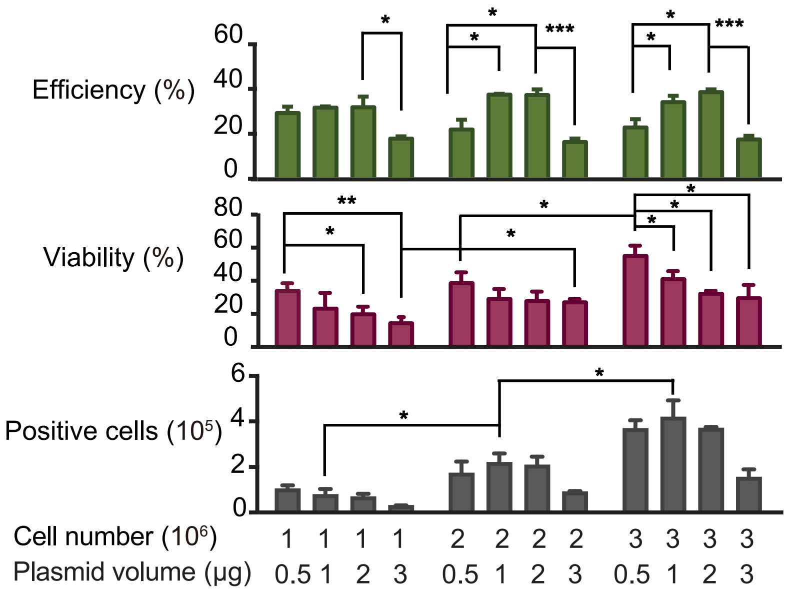

Supplementary Fig. 1 Electroporation of re-stimulated T cells at 500 V. Positive cell number = percent of positive cells × viable cells number.

Supplementary Fig. 1 Electroporation of re-stimulated T cells at 500 V. Positive cell number = percent of positive cells × viable cells number.
