# Supplementary figures and images for: Optimized DNA electroporation for primary human T cell engineering
Source: BMC Biotechnol. 2018 Jan 30;18:4. doi: 10.1186/s12896-018-0419-0 (PMC5789706; doi:10.1186/s12896-018-0419-0)

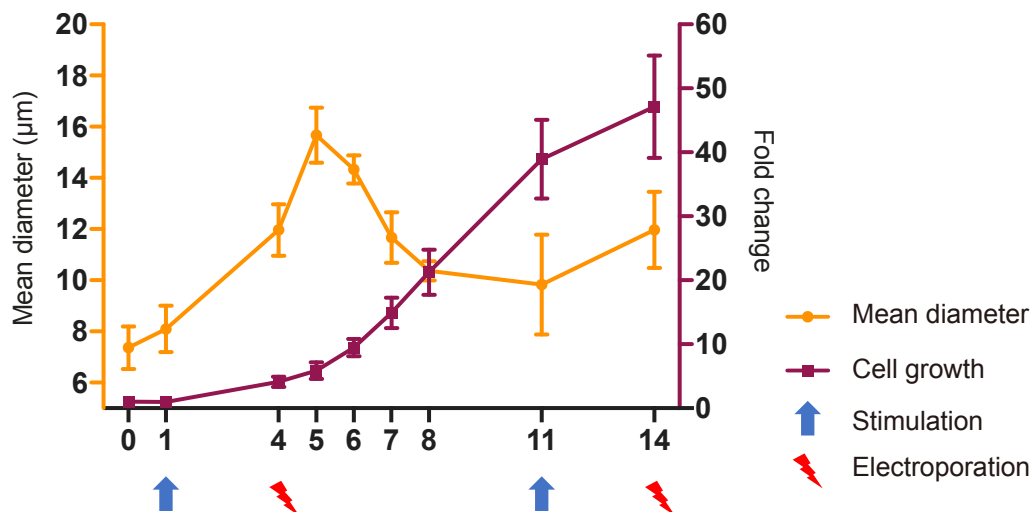

**Supplementary Fig. 2** Changes in the mean diameter and the growth of T cells after activation.

Supplement: Supplementary file 2 — Figure S2. Changes in the mean diameter and the growth of T cells after activation. (PDF 736 kb) [file 12896_2018_419_MOESM2_ESM.pdf]
