# Supplementary material for: Optimized DNA electroporation for primary human T cell engineering
Source: BMC Biotechnol. 2018 Jan 30;18:4. doi: 10.1186/s12896-018-0419-0 (PMC5789706; doi:10.1186/s12896-018-0419-0)
Supplement: Supplementary file 3 — Figure S3. Transgene expression and proliferation of CAR-T cells. (PDF 793 kb) [file 12896_2018_419_MOESM3_ESM.pdf]

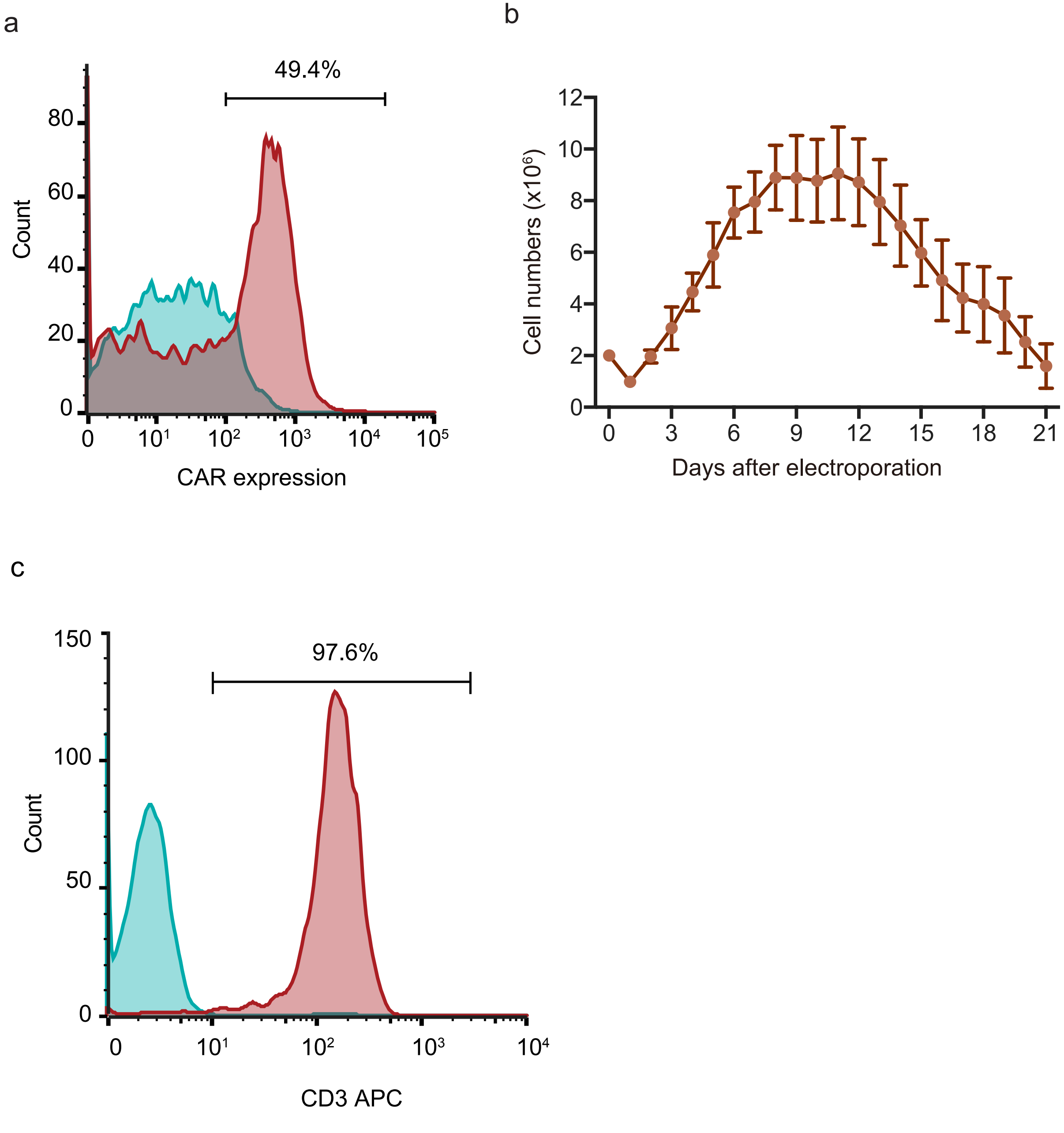

Supplementary Fig. 3 Transgene expression and proliferation of CAR-T cells.
a Red peak, electro-transfected T cells; green peak, untreated T cells. **b** Proliferation of CAR-T cells.
c Red peak, APC anti-human CD3 stained cells; green peak, isotype control stained cells.
