# Supplementary material for: Optimized DNA electroporation for primary human T cell engineering
Source: BMC Biotechnol. 2018 Jan 30;18:4. doi: 10.1186/s12896-018-0419-0 (PMC5789706; doi:10.1186/s12896-018-0419-0)
Supplement: Supplementary file 4 — Figure S4. Change of T cell phenotype after electroporation, initial stimulation, and re-stimulation. (PDF 1080 kb) [file 12896_2018_419_MOESM4_ESM.pdf]

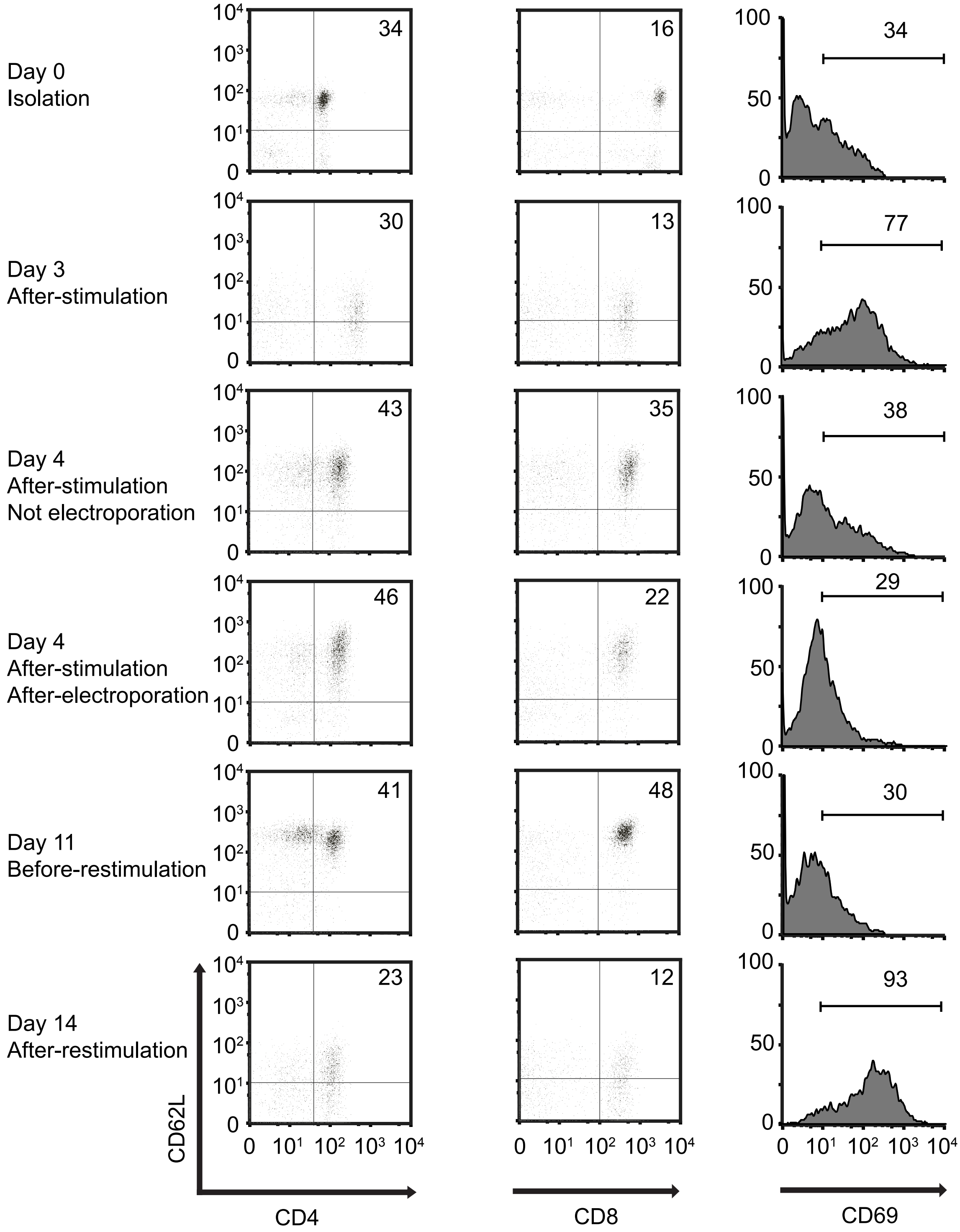

Supplementary Fig. 4 Change of T cell phenotype after electroporation, initial stimulation and re-stimulation. Numbers in dot plot and histogram represent the percent of corresponding regions.
